# Multi-tool copy number detection highlights common body size-associated variants in miniature pig breeds from different geographical regions

**DOI:** 10.1186/s12864-025-11446-8

**Published:** 2025-03-22

**Authors:** Jan Berghöfer, Nadia Khaveh, Stefan Mundlos, Julia Metzger

**Affiliations:** 1 Max Planck Institute for Molecular Genetics, Berlin, Germany; 2 Institute of Chemistry and Biochemistry, Department of Biology, Chemistry and Pharmacy, Freie Universität Berlin, Berlin, Germany; 3 Institute of Animal Genomics, University of Veterinary Medicine Hanover, Hanover, Germany; 4 Institute for Medical and Human Genetics, Charité Universitätsmedizin Berlin, Berlin, Germany; 5 Charité - Universitätsmedizin Berlin, BCRT - Berlin Institute of Health Centre for Regenerative Therapies, Berlin, Germany

**Keywords:** Copy number variation, Pig genome, Next-generation sequencing, Miniature pigs, Quantitative trait loci, Functional enrichment, Regional adaptation, Genetic diversity, Biomedical research

## Abstract

**Background:**

Copy number variations (CNVs) represent a common and highly specific type of variation in the genome, potentially influencing genetic diversity and mammalian phenotypic development. Structural variants, such as deletions, duplications, and insertions, have frequently been highlighted as key factors influencing traits in high-production pigs. However, comprehensive CNV analyses in miniature pig breeds are limited despite their value in biomedical research.

**Results:**

This study performed whole-genome sequencing in 36 miniature pigs from nine breeds from America, Asia and Oceania, and Europe. By employing a multi-tool approach (CNVpytor, Delly, GATK gCNV, Smoove), the accuracy of CNV identification was improved. In total, 34 homozygous CNVs overlapped with exonic regions in all samples, suggesting a role in expressing specific phenotypes such as uniform growth patterns, fertility, or metabolic function. In addition, 386 copy number variation regions (CNVRs) shared by all breeds were detected, covering 33.6 Mb (1.48% of the autosomal genome). Further, 132 exclusive CNVRs were identified for American breeds, 47 for Asian and Oceanian breeds, and 114 for European breeds. Functional enrichment analysis revealed genes within the common CNVRs involved in body height determination and other growth-related parameters. Exclusive CNVRs were located in the region of genes enriched for lipid metabolism in American minipigs, reproductive traits in Asian and Oceanian breeds, and cardiovascular features and body height in European breeds. In the selected groups, quantitative trait loci associated with body size, meat quality, reproduction, and disease susceptibility were highlighted.

**Conclusion:**

This investigation of the CNV landscape of minipigs underlines the impact of selective breeding on structural variants and its role in the development of specific breed phenotypes across geographical areas. The multi-tool approach provides a valuable resource for future studies on the effects of artificial selection on livestock genomes.

**Supplementary Information:**

The online version contains supplementary material available at 10.1186/s12864-025-11446-8.

## Background

Miniature pigs, extremely popular for their small stature and similarities to human physiology, have become indispensable models in biomedical research. However, the genetic background of their distinct phenotypic traits, including the role of copy number variations (CNVs), remains insufficiently characterised. Indeed, mammalian genomes harbour a remarkable diversity of genetic variants that underly breed- or population-specific phenotypes [1]. They range from single-nucleotide polymorphisms (SNPs) and small insertions or deletions (INDELs) to larger structural alterations [2–4]. Among these structural variants, CNVs are crucial for both natural and artificial selection [5]. CNVs can be duplicated or deleted elements, ranging from 50 base pairs (bp) to several megabase pairs [6–9]. They can influence gene expression by altering the copy number of genes and regulatory elements, affecting cellular function and phenotypic traits [10–12].

During evolution and subsequent domestication of animals, CNVs have been crucial in introducing genetic diversity and supporting the selection of desirable traits such as improved disease resistance, growth rate, and productivity [13–17]. The interplay between natural and artificial selection on specific CNVs has guided evolutionary trajectories in both wild and domesticated populations [18–20].

Various methods have been employed to detect CNVs, including SNP microarrays, array-based Comparative Genomic Hybridisation (aCGH), and Whole-Genome Sequencing (WGS) [21–23]. Though microarrays are widely used, WGS allows more accurate CNV detection due to their high resolution [24–26]. Recent advances in bioinformatics have facilitated the identification of small CNVs from short-read sequencing data despite difficulties arising from complex or repetitive genomic regions [27, 28]. In contrast, long-read sequencing technologies have been proposed to be advantageous for resolving complex structural variants. Their application in livestock genomics remains limited due to high costs and computational demands [27–29]. Consequently, short-read sequencing remains the primary method for large-scale CNV studies in domestic animal populations [26].

To enhance sensitivity and precision in short-read WGS-based CNV analysis, combining multiple CNV tools has been highlighted as an effective strategy [5, 16, 30]. This multi-tool approach captures a broader spectrum of variation by integrating signals from paired-end reads, split reads, or coverage depth [31, 32]. The annotation of resulting data remains limited but could be improved with more widespread use of WGS [5].

Recent livestock studies have taken advantage of these improved methods and demonstrated the significant impact of CNVs on various phenotypic traits [16, 33, 34]. For instance, a CNV in the *eukaryotic translation initiation factor 4A2 (EIF4A2)* gene was linked to hip width, rump length, heart girth, and chest depth in several Chinese cattle breeds [35]. Furthermore, a CNV-based genome-wide association study (GWAS) in horses revealed three deleted CNV regions (CNVRs) associated with larger body sizes [16, 17, 36, 37]. In pigs, a comprehensive screening of CNVs across 18 diverse populations identified candidate copy number variable genes associated with complex traits [38]. Additionally, reproductive traits have been analysed for significant CNVs in pigs [39, 40], showing, for example, associations with litter size and teat number in Bama Xiang pigs [39]. Moreover, recent studies highlighted CNVs acting as modifiers for intramuscular fat content [41–43]. However, most CNV studies in pigs predominantly focused on traditional and commercial breeds commonly used in agriculture [17, 36, 37].

In addition, CNVs have been studied to identify the evolutionary genomic differences between domestic pigs and their wild ancestor [17, 44]. A comparison of the CNV-landscape between Anqingliubai pigs and Asian wild boars revealed genes related to growth (*CD36*), reproduction (*CIT*,* RLN*), detoxification (*CYP3A29*), and fatty acid metabolism (*ELOVL6*) [44]. Furthermore, genome sequence diversity has been put into context with phenotypic and production traits of Chinese and Western breeds, as well as with hypoxia and body size for Chinese native Tibetan, Dahe, and Wuzhishan pigs [45, 46]. Particular attention may be given to laboratory minipig herds to maintain genetic diversity and avoid the accumulation of fitness-reducing mutations [47].

Miniature pigs represent individuals with particularly small body sizes and distinct disease resistance profiles, making them suitable for biomedical research [48, 49]. Due to their physiological and anatomical similarities to humans, miniature pigs serve as valuable models for cardiovascular, diabetes, or obesity research and xenotransplantation [50–52]. Understanding copy number variability in miniature pigs could clarify the mechanisms underlying their unique phenotypic characteristics and reinforce their importance as large animal models in research [39, 53, 54].

The present study provides comprehensive insights into the CNV landscape and genetic diversity among miniature pig breeds, using a multi-tool approach for CNV detection. These findings improve our understanding of the genetic basis of phenotypic traits, enhance their utility as biomedical research models, and support more effective breeding strategies.

## Results

### CNVs discovery and distribution

**Fig. 1:**
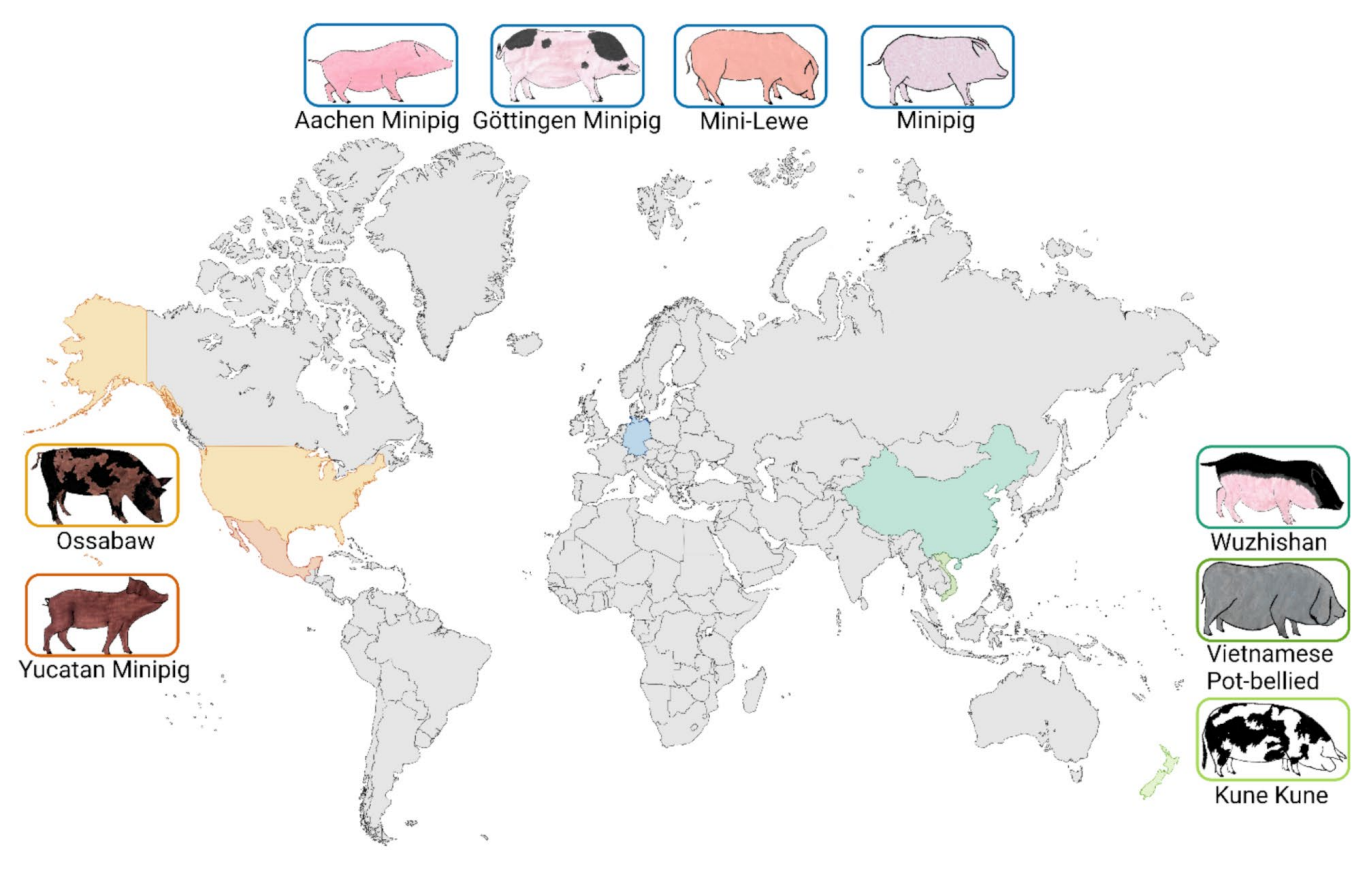
Overview of the geographical origins and phenotypes. The phenotypes and geographical origins of the nine analysed miniature pig breeds from America (orange: Mexico and USA), Asia and Oceania (green: China, Vietnam, and New Zealand), and Europe (blue: Germany) are displayed. The figure was created with BioRender.com

WGS data from 36 miniature pigs representing nine breeds from different geographical origins worldwide were analysed to test the assumption that miniature pig breeds harbour CNVRs associated with their specific regional phenotype, as well as CNVRs common in all miniature pig breeds, regardless of their geographical origin (Fig. 1). Quality metrics from short-read WGS alignments revealed a mean alignment rate of 99.63% and mean genome coverage of 99.45%, reflecting a broad and consistent genome representation. The mean sequencing depth of 36.75X (minimum depth 14.06X) provided a resolution suitable for accurate detection of CNVs, including smaller variants (Additional file 1: Table S1). A summary of the number of CNVs identified by each tool (CNVpytor, Delly, GATK gCNV, and Smoove) based on CNV type (gain, loss or a mix of both) and the distribution of the CNV length per breed is presented in Additional file 2: Table S2. The distribution of CNV lengths showed most CNVs within a small range, with a sharp decline in frequency with increasing CNV size (Additional file 3: Figure S1). Among the tools, CNVpytor identified the highest number of CNVs; notably, all four tools detected significantly more losses than gains. Based on the detected CNVs, multidimensional scaling (MDS) of the nine miniature pig breeds showed an apparent clustering of individuals by breed, with distinct groupings for European, Asian, and American breeds (Additional file 4: Figure S2). Similarly, the neighbour-joining (NJ) tree reflected these genetic separations, showing consistent clustering of individuals within breeds and highlighting their distinct genomic profiles and breeding history shaped by geographical origin and selective breeding practices.

To explore CNVs with potential functional impacts, filters were applied for homozygous CNVs and multi-copy regions (copy numbers > 4) overlapping with exons in all 36 minipig samples. 34 CNVs met these criteria in all samples, comprising 27 losses (deletions) and seven gains (duplications). These CNVs overlapped with and potentially affected 195 exonic regions corresponding to 64 genes (Additional file 5: Table S3), including *pregnancy-associated glycoprotein 6 (PAG6)*, *chromodomain helicase DNA binding protein 1 (CHD1L)*,* cadherin EGF LAG seven-pass G-type receptor 1 (CELSR1)*,* early endosome antigen 1 (EEA1)*,* cytochrome P450 family 4 subfamily A member 24 (CYP4A24)*,* collagen type VI alpha 1 chain (COL6A1)*,* phospholipase C delta 4 (PLCD4)* and *RPTOR independent companion of MTOR complex 2 (RICTOR)*. These loci might be involved in different biological processes, such as reproduction, metabolic regulation, and tissue development, supporting the relevance of these structural variants in shaping breed-specific traits.

The validation of seven selected CNVs overlapping with exonic regions by real-time quantitative PCR (qPCR) confirmed the losses or gains in the miniature pigs (Additional file 6: Table S4). Among five entirely randomly selected CNVs across different chromosomes, two regions with predicted losses and one region with a predicted gain could be validated, whereas two predicted gains could not be confirmed.

### CNVRs shared by all minipig populations and regionally grouped populations

**Fig. 2:**
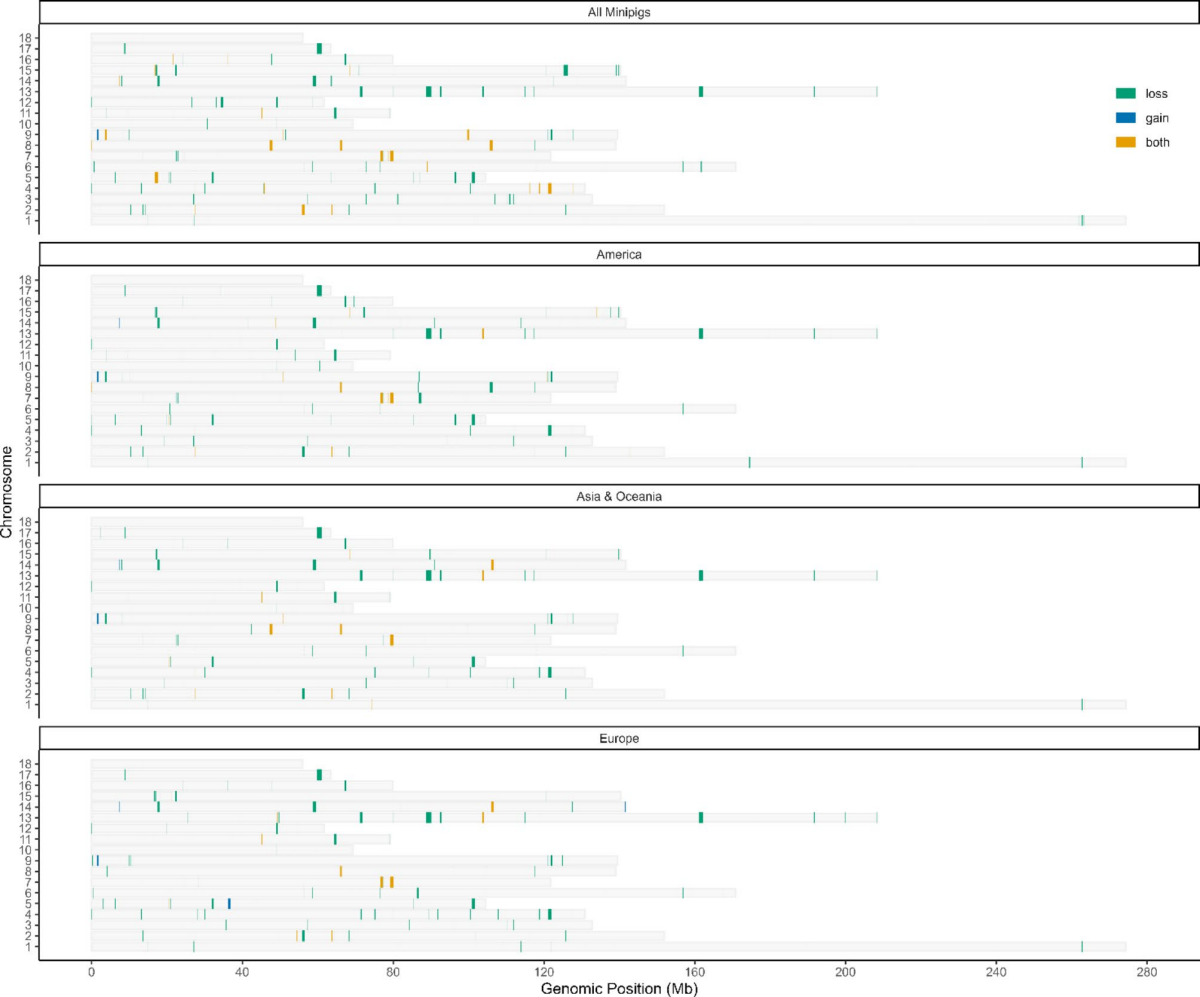
Distribution of shared minipig-CNVRs across all nine global breeds and by regional breed groups. Grey bars represent all 18 pig autosomes. Green segments indicate deleted ‘loss’ CNVRs, while blue segments highlight duplicated ‘gain’ CNVRs. Orange segments show ‘both’ CNVRs, which contain regions with both ‘loss’ and ‘gain’. The graph is divided into four panels: one for all minipig breeds combined (All Minipigs) and three for regional breed groups (America, Asia & Oceania, Europe). Note that only large CNVRs are visible in this graph

After calling and filtering the CNVs, genome-wide CNVRs were assembled, and the chromosomal distribution of CNVRs and their types (gain, loss or both) were summarised (Additional file 7: Table S5). A total of 386 CNVRs were identified among CNVRs shared by at least two individuals from all nine miniature pig breeds distributed across different chromosomes (Fig. 2, see also Additional file 8: Figure S3 for a detailed view in an ideogram). These CNVRs included five copy number gains, 345 copy number losses, and 36 CNVRs with a mix of both types.

The sizes of the CNVRs ranged from 180 to 1,330,687 bp, with an average of 87,057.15 bp and a median of 559 bp. The sum of CNVRs occupied 33,604,061 bp, representing 1.48% of the autosomal *Sus scrofa 11.1* genome. Smaller CNVRs were more frequent, which aligns with prior observations that shorter structural variants are less likely to undergo purifying selection (Additional file 9: Figure S4). The number of CNVRs on each chromosome ranged from five on chromosome 18 to 34 on chromosome 2 (Additional file 10: Table S6) and was relative to the chromosomal length (Additional file 9: Figure S4). Taken together, these shared CNVRs underscore the presence of deletions across all minipig breeds and highlight the genome-wide prevalence of smaller variants with potential functional relevance.

### Comparison of CNVRs across regionally grouped populations

Subsequent analysis of the shared CNVRs resulted in 430 CNVRs in all ten individuals of the two miniature pig breeds from America (Ossabaw and Yucatan minipig, Additional file 7: Table S5). These CNVRs included nine copy number gains, 398 copy number losses, and 23 CNVRs with a mixture of both types (Fig. 2). The sizes of the CNVRs ranged from 194 to 1,330,687 bp, with a mean of 58,773.94 bp and a median of 597 bp. The CNVRs occupied 25,272,793 bp, representing 1.12% of the autosomal genome. Shorter CNVRs were more abundant than longer CNVRs in the genome (Additional file 9: Figure S4). The number of CNVRs on each chromosome ranged from three on chromosome 18 to 36 on chromosome 5, increasing with chromosome length (Additional file 10: Table S6; Additional file 9: Figure S4).

For the three miniature pig breeds from Asia and Oceania (Kune Kune, Vietnamese Potbellied Pig, and Wuzhishan Minipig), 382 CNVR were identified that are shared by all nine individuals (Additional file 7: Table S5). These CNVRs included seven copy number gains, 354 copy number losses, and 21 CNVRs with a mixture of both types (Fig. 2). The sizes of the CNVRs ranged from 191 to 1,330,687 bp, with an average of 63,869.72 bp and a median of 502 bp. The collective length of the CNVRs was 24,398,232 bp, representing 1.08% of the autosomal genome. The CNVRs identified on each chromosome ranged from five on chromosome 18 to 32 on chromosomes 2 and 8 (Additional file 10: Table S6). Shorter CNVRs were more prevalent than longer CNVRs in the genome, and the number of detected CNVRs increased with increasing chromosome length (Additional file 9: Figure S4).

Furthermore, for the four miniature pig breeds from Europe (Aachen Minipig, Göttingen Minipig, and Mini-Lewe Minipig; Additional file 7: Table S5), 388 CNVRs were called that are shared by all 17 individuals, including ten copy number gains, 360 copy number losses, and 18 CNVRs with a combination of both types. The sizes of the CNVRs ranged from 191 to 1,330,687 bp, with an average of 69,129.91 bp and a median of 564.5 bp. The collective length of the CNVRs was 26,777,008 bp (1.18% of the autosomal genome). The number of CNVRs identified on each chromosome ranged from three on chromosome 18 to 34 on chromosome 4 (Additional file 10: Table S6). Consistent with the other regional groups, shorter CNVRs were more frequent, and their chromosomal distribution correlated with chromosome length. Altogether, these findings highlight that each regional group of minipigs exhibits a distinct but overlapping set of CNVRs.

### Functional enrichment of genes overlapping with common CNVRs

For functional enrichment analysis, 359 genes (no duplicates) were extracted from 370 CNVRs shared by at least two individuals across all nine miniature pig breeds (see Additional file 7: Table S5). In addition, for the regional subsets, we identified 380 genes from 430 shared CNVRs (America), 357 genes from 382 shared CNVRs (Asia and Oceania), and 358 genes from 388 shared CNVRs (Europe; Additional file 7: Table S5).

**Fig. 3:**
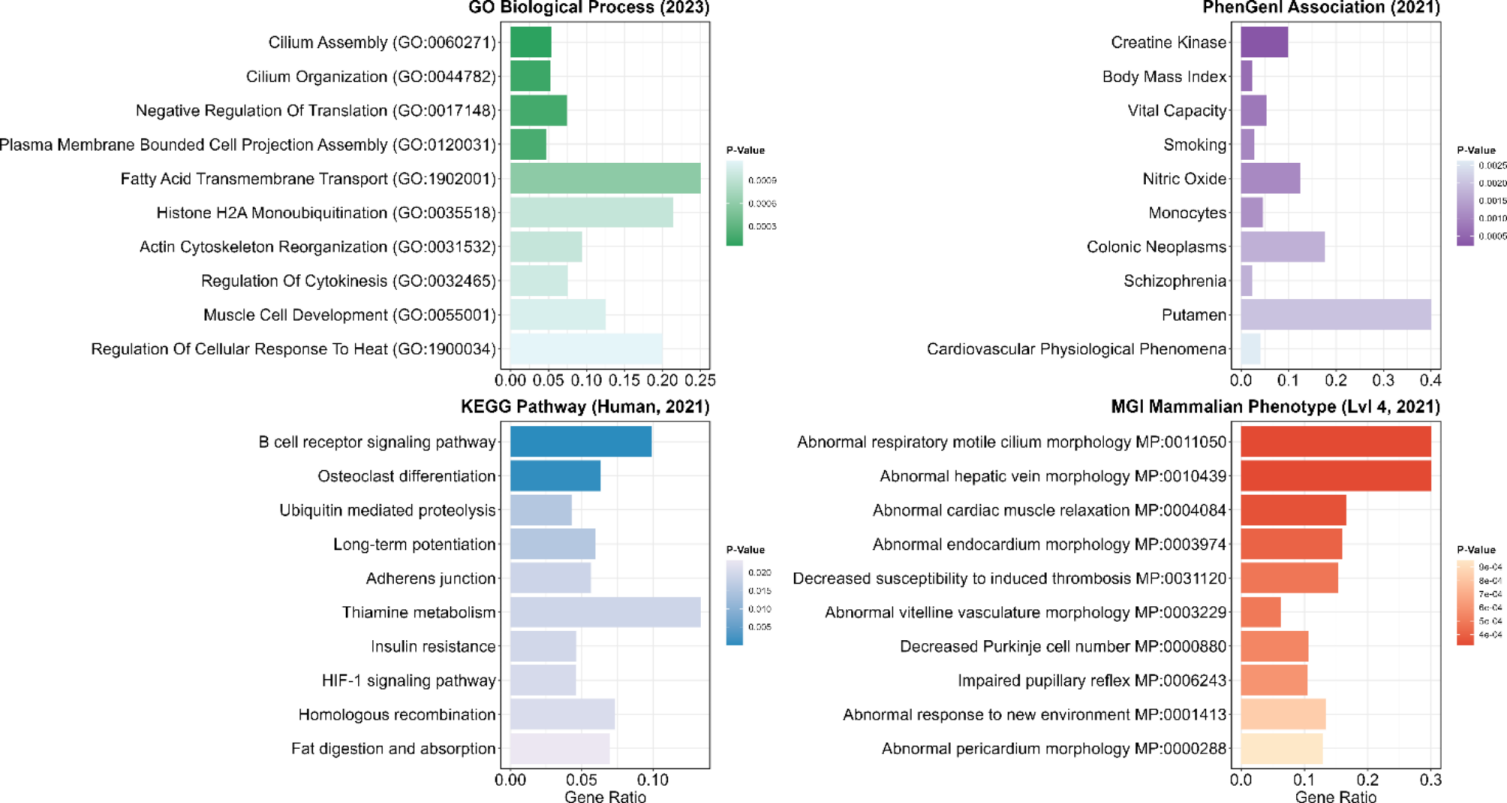
Functional enrichment of genes in CNVRs detected in all nine minipig breeds. The illustration depicts the ten most significantly enriched GO terms from the databases “GO_Biological_Process_2023”, “KEGG_2021_Human”, “MGI_Mammalian_Phenotype_Level_4_2021”, and “PhenGenI_Association_2021”. The terms are ordered by *p*-value (colour intensity) and represent CNVR genes common to all nine minipig breeds. The X-axis indicates the gene ratio

Gene set enrichment analysis of 286 human orthologous genes from 359 pig genes in CNVRs shared by all minipig breeds revealed significantly enriched Gene Ontology (GO) biological processes, including cilium assembly (GO:0060271), fatty acid transmembrane transport (GO:1902001), actin cytoskeleton reorganisation (GO:0031532), and muscle cell development (GO:0055001) (Fig. 3; Additional file 11: Table S7). Pathways from the Kyoto Encyclopaedia of Genes and Genomes (KEGG), such as B-cell receptor signalling, osteoclast differentiation, insulin resistance, and fat digestion and absorption, were also enriched, along with terms like body mass index and monocytes from the PhenGenI association database. Among these, 46 significantly enriched genes, such as *CPNE8*, *CDH4*, and *INSR*, were linked to the PhenGenI term “body mass index, whereas 34 genes, including *DPP10*,* CLSTN2*, and *ADAMTS17*, were associated with the term “height”. In addition, the MGI Mammalian Phenotypes database highlighted enriched terms related to respiratory motility (MP:0011050), hepatic vein morphology (MP:0010439), cardiac muscle relaxation (MP:0004084), and responses to novel environments (MP:0001413).

### Functional enrichment of genes overlapping with regionally grouped CNVRs

**Fig. 4:**
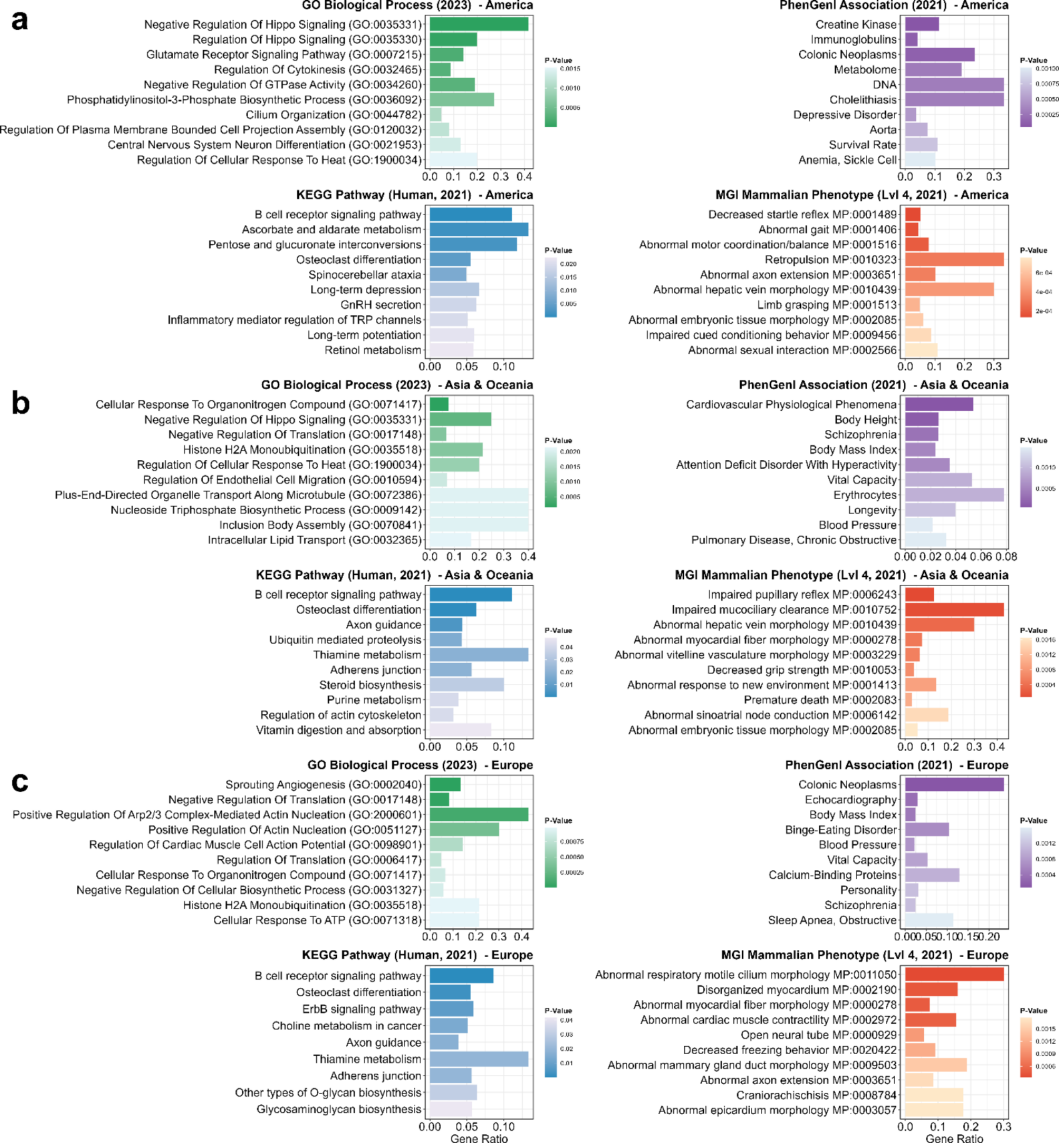
Functional enrichment analysis for genes in CNVRs called for regionally grouped minipig breeds. The figure illustrates the ten most significantly enriched terms for the databases “GO_Biological_Process_2023”, “KEGG_2021_Human”, “MGI_Mammalian_Phenotype_Level_4_2021”, and “PhenGenI_Association_2021”. The data set comprising CNVR genes common to all regionally grouped breeds originating from America (**a**), Asia and Oceania (**b**) or Europe (**c**) has been sorted by *p*-value (colour intensity). The X-axis indicates the gene ratio

Analogous to the complete dataset, functional enrichment was also performed for CNVRs identified in pigs from distinct geographical regions (America, Asia & Oceania, Europe, see Fig. 4). These analyses included 315 human orthologous genes from American breeds (out of 380 pig CNVR-genes), 292 genes from Asian and Oceanian breeds (357 pig CNVR-genes) and 290 genes from European breeds (358 pig CNVRs-genes; Additional file 11: Table S7).

### Functional enrichment in American miniature pig breeds

Genes in CNVRs shared by the two American breeds were significantly enriched for GO processes, such as negative regulation of Hippo signalling (GO:0035331), regulation of Hippo signalling (GO:0035330), glutamate receptor signalling pathway (GO:0007215), and negative regulation of GTPase activity (GO:0034260) (Fig. 4a). Enriched KEGG pathways included the B-cell receptor signalling pathway, ascorbate and aldarate metabolism, pentose and glucuronate interconversions, and osteoclast differentiation. In addition, the PhenGenI association highlighted significantly enriched terms such as creatine kinase and metabolome. The MGI Mammalian Phenotypes indicated traits like abnormal gait (MP:0001406) and abnormal hepatic vein morphology (MP:0010439).

### Functional enrichment in Asian and Oceanian miniature pig breeds

For genes in CNVRs shared by three breeds from Asia and Oceania, functional gene set enrichment analysis revealed significant GO terms such as cellular response to organonitrogen compound (GO:0071417), negative regulation of Hippo signalling (GO:0035331), negative regulation of translation (GO:0017148), and histone H2A monoubiquitination (GO:0035518) (Fig. 4b). Significant KEGG pathways resembled those in American minipigs (e.g. B-cell receptor signalling; osteoclast differentiation), whereas PhenGenI associations featured cardiovascular traits, body height, body mass index, and vital capacity. Furthermore, the MGI Mammalian Phenotypes revealed enrichments related to sinoatrial node conduction (MP:0006142), hepatic vein morphology (MP:0010439), and myocardial fibre morphology (MP:0000278).

### Functional enrichment in European miniature pig breeds

For genes in CNVRs shared by four breeds from Europe, several GO biological processes were enriched, including sprouting angiogenesis (GO:0002040), negative regulation of translation (GO:0017148), positive regulation of actin nucleation (GO:0051127), and regulation of cardiac muscle cell action potential (GO:0098901) (Fig. 4c). KEGG pathways included B-cell receptor signalling, osteoclast differentiation, ErbB signalling pathway, and choline metabolism in cancer. The PhenGenI association identified significantly enriched terms such as body mass index and vital capacity, and the MGI Mammalian Phenotypes showed significantly enriched terms, such as abnormal respiratory motile cilium morphology (MP:0011050), disorganised myocardium (MP:0002190), abnormal myocardial fibre morphology (MP:0000278), and abnormal cardiac muscle contractility (MP:0002972).

### Enrichment of QTL overlapping with common CNVRs

**Fig. 5:**
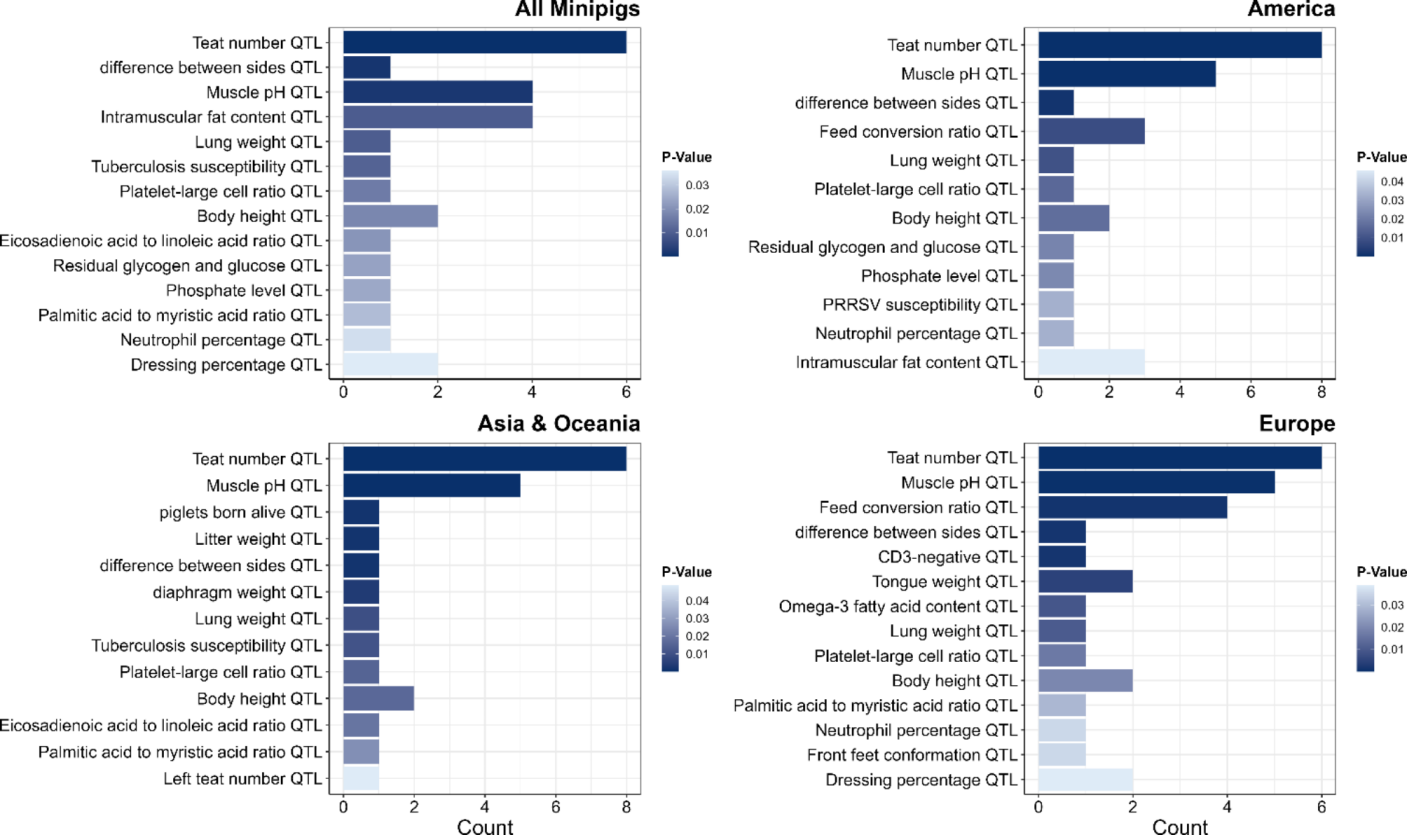
Enrichment analysis of QTL overlapping with CNVRs. Enrichment analysis was conducted for significant QTL found in the same regions overlapping with CNVRs shared by all nine miniature pig breeds or for regionally grouped minipig breeds (America, Asia & Oceania, or Europe). The figure shows all significantly enriched QTL in the CNVRs compared to the autosomal genome in total. The dataset comprising QTL common to all nine minipig breeds (**a**) or the common regionally grouped breeds originating from America (**b**), Asia and Oceania (**c**), and Europe (**d**) was sorted by *p*-values

As an additional functional evaluation of CNVRs, an overlap analysis was performed with quantitative trait loci (QTL) from the animal QTL database (Fig. 5; Additional file 12: Table S8). Among 31,312 QTL examined, 52 were associated with 36 phenotypic traits and overlapped with CNVRs shared by all nine minipig breeds. QTL enrichment analysis revealed that approximately 39% of these traits were significantly overrepresented (*p* < 0.05). These traits included body height, difference between two sides, muscle pH QTL, intramuscular fat content QTL, tuberculosis susceptibility QTL, and several haematological and metabolic QTL. Interestingly, the analysis revealed a significantly enriched height QTL within two common CNVRs shared by all nine minipig breeds. One overlapped with the *chromodomain helicase DNA binding protein 4* (*CHD4*) gene.

In addition, QTL overlap was tested for the regional breed groups. In the American breeds, 50 QTL were associated with 34 phenotypic traits, of which 12 (35%) showed significant overrepresentation. These traits were mainly related to the QTL body height, difference between sides, muscle pH QTL, intramuscular fat content QTL, PRRSV susceptibility, and haematological or metabolic parameters. In Asian and Oceanian breeds, 49 QTL representing 36 phenotypic traits overlapped with CNVRs, of which 13 (36%) were significantly overrepresented, suggesting an emphasis on body size, reproduction, and meat quality. In European minipigs, 54 QTL coincided with 37 phenotypic traits, of which 14 (38%) were significantly overrepresented, reflecting an association with body height, conformation, meat quality, disease susceptibility, and immunity. Overall, these region-specific QTL overlaps and the globally shared patterns indicate that CNVs are integral to phenotypic diversity in minipigs worldwide, mainly for growth, disease resistance, and production traits.

### Exclusive CNVRs across regionally grouped populations

**Fig. 6:**
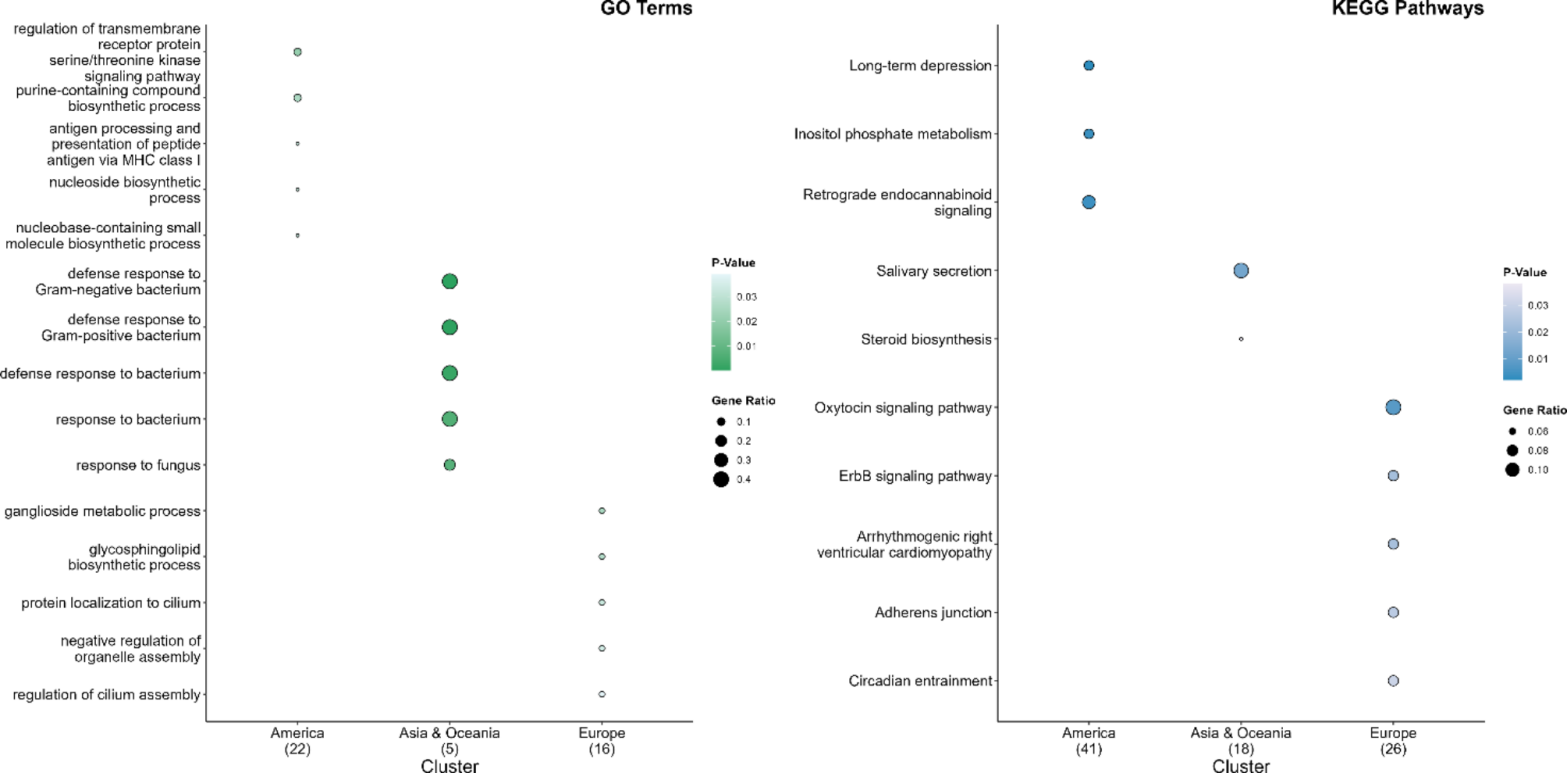
Biological theme comparison of genes overlapping with exclusive CNVRs in regionally grouped minipig breeds. Biological themes were compared for genes overlapping with exclusive CNVRs identified in all regionally grouped minipig breeds (America, Asia, Oceania, and Europe). The figure illustrates the five most significantly enriched Gene Ontology (GO, left) biological processes and KEGG term clusters (right). On the x-axis, each geographic region (America, Asia & Oceania, Europe) and the number of annotated genes are indicated (in parentheses). The *p*-value is displayed by colour intensity, and the dot size indicates the gene ratio for each cluster

Finally, CNVRs uniquely detected in each regional group (named exclusive CNVRs) were investigated. Overall, 132 exclusive CNVRs were identified in two miniature pig breeds from America, harbouring one gain, 125 losses, and six CNVRs with a mixed gain-and-loss pattern (Additional file 13: Table S9). Their lengths ranged from 237 to 649,999 bp (average size: 23,651.70 bp, median: 1120 bp), encompassing 0.14% of the autosomal genome (3,122,025 bp). In the three miniature pig breeds from Asia and Oceania, 47 exclusive CNVRs were detected, comprising 45 losses and two mixed types with an average size of 21,302.47 bp (median: 661 bp). These regions covered 0.04% of the autosomal genome (1,001,216 bp). European minipigs (four breeds) exhibited 114 exclusive CNVRs with three copy number gains, 107 losses, and four mixed types. The lengths ranged from 217 to 585,179 bp (mean: 47,224.74 bp; median: 1629.5 bp), covering 0.24% of the autosomal genome (5,383,620 bp).

Finally, we compared biological themes to search for enriched functional profiles for the genes within the unique CNVRs. Genes were grouped according to their geographical origin: 125 genes from America, 47 from Asia and Oceania, and 109 from Europe (Additional file 14: Table S10). The analysis revealed several significantly enriched functional clusters of GO biological processes and KEGG terms for each regional gene set that overlapped with exclusive CNVRs (Fig. 6). The American gene set showed many terms on metabolic regulation, including “nucleobase-containing small molecule biosynthetic process” (GO:0034404) and other biosynthetic pathways. In contrast, gene sets from Asia and Oceania highlighted “immune responses to bacterial and fungal pathogens”, suggesting specialisation in immune defence systems. The European gene set showed enrichment in ciliary function, organelle assembly, and lipid metabolism (e.g. glycosphingolipid biosynthesis), reflecting distinct local adaptations. Further analysis of KEGG pathways emphasised neurological and metabolic signalling in American minipigs, a more specialised immune response profile in Asia and Oceania, and cardiovascular and circadian regulatory adaptations in Europe. These region-specific enrichment patterns indicated exclusive CNVRs harboured genes under potential selection in local breeding histories.

### Distribution of QTL overlapping exclusive CNVRs

In addition, an overlap of exclusive CNVRs with QTL assessed for regionally grouped minipig breeds revealed distinct patterns in America, Asia and Oceania, and Europe (Table 1). In the American breeds, 15 QTL corresponding to 11 phenotypic traits overlapped with exclusive CNVRs, encompassing growth and developmental characteristics such as teat number, feed conversion ratio, birth weight variability, and longissimus muscle area, in addition to meat and carcass quality traits (“muscle pH”, “intramuscular fat content”, “stearic acid content”), reproduction (“sperm abnormality” and “stillbirth rates”), and a health-immunity QTL for “PRRSV susceptibility”. In Asia and Oceania, four QTL were identified, reflecting a narrower range of traits associated primarily with growth (“teat count”, “diaphragm weight”) and reproduction (“litter weight”, “live-born piglets”). By contrast, European minipig breeds exhibited a more extensive distribution, with 16 QTL linked to 15 phenotypic traits, including growth and development, meat and carcass quality, health and immunity, and reproduction. Noteworthy traits included “body height”, “shoulder weight”, “meat colour”, and immune-related markers such as “CD3-negative” and “CD8-negative” or “CD8-positive leukocyte percentage”. Altogether, these region-specific patterns underscore that exclusive CNVRs are closely tied to traits of regional importance, reflecting distinctive breeding histories and local adaptations.

**Table 1:** Distribution of QTL overlapping exclusive CNVRs in regionally grouped minipig breeds. The table summarises all QTL overlapping with exclusive CNVRs identified for all regionally grouped minipig breeds (America, Asia & Oceania, and Europe) assigned to the functional categories as ‘growth and development’, ‘reproduction’, ‘meat and carcass quality’ and ‘health and immunity’ to provide insights into region-specific trait association

Category	QTL-Counts	America	Asia & Oceania	Europe
Growth and Development	Teat number	5	1	1
	Feed conversion ratio	1	0	2
	Birth weight variability	1	0	0
	Body height	0	0	1
	Diaphragm weight	0	1	0
	Longissimus muscle area	1	0	0
	Shoulder weight	0	0	1
	Subcutaneous fat thickness	1	0	0
	Tongue weight	0	0	1
	**Total QTL**	**9**	**2**	**6**
Health and Immunity	CD3-negative	0	0	1
	CD8-negative leukocyte percentage	0	0	1
	CD8-positive leukocyte percentage	0	0	1
	Front feet conformation	0	0	1
	PRRSV susceptibility	1	0	0
	**Total QTL**	**1**	**0**	**4**
Meat and Carcass Quality	Muscle pH	1	0	1
	Cis-11-Eicosenoic acid to oleic acid ratio	0	0	1
	Intramuscular fat content	1	0	0
	Meat colour	0	0	1
	Omega-3 fatty acid content	0	0	1
	Palmitoleic acid to palmitic acid ratio	0	0	1
	Stearic acid content	1	0	0
	**Total QTL**	**3**	**0**	**5**
Reproduction	Sperm abnormality rate	1	0	1
	Litter weight	0	1	0
	Number of stillborn	1	0	0
	Piglets born alive	0	1	0
	**Total QTL**	**2**	**2**	**1**

## Discussion

This study presents a comprehensive analysis of the genomic CNV landscape of nine miniature pig breeds from diverse geographical regions using a multi-tool approach. By integrating multiple CNV detection methods, both common and exclusive CNVRs were identified, offering valuable insights into genetic diversity, regional adaptability, and possible implications for breeding strategies.

Employing a multi-tool approach likely increased the sensitivity and accuracy of CNV identification compared to traditional single-tool methods. In particular, the validation of those CNVs was conducted using a minimum of two tools, confirming the in-silico approach’s robustness. By integrating detection methods based on read depth, split reads, and paired-end reads, a broader range of CNVs could be captured, including smaller and more complex variants, which might otherwise remain undetected [5, 30]. This finding is consistent with a previous study, suggesting that the combination of tools represents a good compromise between specificity and sensitivity for calling CNVs of different types in short-read sequencing data [5]. Nevertheless, the detection efficiency is still highly dependent on the read length: longer reads tend to reduce mismatches and multiple alignments [55], whereas shorter reads (> 100 bp) can present challenges in resolving variant-dense regions [55, 56]. Although long-read sequencing technologies help overcome some complexities in repetitive genomic regions [57], their higher costs and computational demands continue to limit their application in livestock genomics.

In practice, both long- and short-read sequencing platforms offer distinct advantages and drawbacks: Long reads can more readily resolve CNVs in regions inaccessible to arrays or short-read data, whereas short-read approaches offer high resolution for smaller private CNV calls [58]. Moreover, both approaches are relatively independent of population-level CNV frequencies compared to SNP arrays. This is particularly advantageous for studies with small sample sizes or livestock breeds with lower genetic diversity, such as pigs [17, 59]. This advantage is enhanced by recent PCR-independent library preparations that deliver uniform signal intensities during sequencing, facilitating sample-specific depth normalisation without batch processing [60, 61]. Additional techniques, such as 10X Genomics linked-read sequencing [62], DNA template strand sequencing [57] and Hi-C [63], also show promise for increasing CNV detection accuracy, though their use remains limited so far.

The multi-tool analysis in this study allowed the detection of relatively large CNVRs of an average size of 87 kb. In contrast, a previous study in 16 wild and domestic high-production pigs - relying on a single (read-depth based) CNV caller - reported a smaller average CNVR size of 13 kb [17]. The detection of larger CNVRs here likely reflects the broader coverage afforded by a multi-tool method. Furthermore, it has been proposed that rare CNVs are usually longer than common CNVs due to purifying selection against large genomic alterations, particularly in variation-sensitive regions [31]. Consistent with this, the present data show that shorter CNVRs occur more frequently than longer ones.

In addition, all four tools detected significantly more losses than gains, similar to what was observed in previous CNV studies [16, 64]. It was suggested that gains and losses might be differently balanced depending on the CNV-detection tool and subsequent detection method [65]. Apart from the technical context, losses are also proposed to be generally more frequent than gains due to their simpler mutational mechanisms, such as unequal crossover or replication slippage, and their lower likelihood of causing gene dosage imbalances [66, 67].

Interestingly, 195 exonic regions overlap with 34 homozygous deletions, duplications, or multi-copy CNVs, suggesting a potential functional impact of these structural variants in the analysed minipig breeds. Several genes appear to underpin key biological processes and might influence traits enhanced by breeding, including growth, reproductive success, and metabolic function [47, 68, 69]. Notably, genes such as *PAG6* involved in maternal recognition of pregnancy, immunomodulation and trophoblast invasion [70, 71], *CELSR1*, linked to teat number in pigs [72–74] and dosage-dependent effects on embryogenesis or craniofacial development [75, 76], as well as *EEA1*, more highly expressed in embryos of low fertility bulls [77], highlighted the potential role of CNVRs in embryonic development and survival of newborn piglets. However, CNVRs in the region of *EEA1* need to be considered with caution as it was previously suggested that the presence of minor CNV in the reference genome assembly, *Sus scrofa 11.1*, might lead to the predictions of an abnormally gained copy number on *EEA1*, as it was observed in multiple domesticated pig populations and wild boars [78].

Furthermore, members of the CYP4A family, including *CYP4A24* detected in our analysis, have been investigated in the context of liver metabolism and meat quality in pigs [79–81]. The intense selection pressure on performance traits such as meat quality and fat content observed in minipigs might be reflected by the CNVRs found in *COL6A1*, a major extracellular matrix component contributing to the structural integrity in connective tissues with a potential effect on muscle quality [82, 83], *CNOT9*, a factor for meat colour traits in commercial pig breeds [84, 85] or *PLCD4*, which was reported to harbour variants affecting feed conversion, intramuscular fat, and water exudation in pigs [86]. Moreover, it was interesting to note that also an exonic region of *RICTOR*, a component of the mTORC2 complex and a potential regulator of cytoskeletal organisation, metabolism and cell growth [87, 88], was highlighted in our results and might subsequently be associated with growth effects in miniature pigs. This outcome of the analysis of CNVs overlapping with exonic regions suggests that dosage effects might control some key genes and are, therefore, potential candidates for future functional investigations.

In addition, 386 CNVRs common to all investigated miniature pig breeds were detected. As anticipated for miniature-sized breeds, our analysis revealed a significant enrichment of body height QTL within two common CNVRs, overlapping with *CDH4*, a major component of the nucleosome remodelling and deacetylase complex [89]. This gene plays an essential role in epigenetic transcriptional repression, highlighting the potential phenotypic impact of changes in the gene regulatory landscape, such as epigenetic mechanisms [90]. Furthermore, gene set enrichment analysis revealed 34 genes in “body height” and 46 genes in “body mass index”, including *CDH4* (PhenGenI Associations), further supporting the hypothesis that structural variants contribute to the small stature targeted in miniature pig breeding. Consequently, these findings align with previous findings from a genome-wide association study across 58 pig populations, highlighting various CNVs significantly associated with body length and weight [91].

Subsequent analysis found QTL for “body height” and “difference between sides” within CNVRs shared by the analysed minipig breeds, implying these as indicators for intense selection pressures for small body size [92], with miniature size preserved proportionally in all body parts [93]. Signatures of selection for this extreme phenotype were recognised in various breeds, including the Yucatan miniature pig, for which genomic regions under potential selection were associated with feed intake, weight regulation, cell cycle and cell proliferation [92, 94]. Genes related to body mass index, fat digestion, muscle cell development, and creatine kinase were also enriched, hinting at traits under selection associated with miniature body size or targeted breeding strategies for animal models in biomedical research [95]. For instance, fatty acid transport and insulin resistance support the metabolic adaptability of minipigs, making them suitable models for obesity research [95, 96].

Beyond these common CNVRs, exclusive regions in American, European, and Asian/Oceanian breeds were discovered. As emphasised by the phylogenetic tree, the minipigs showed a clustering pattern according to their geographical origins and breeding history, suggesting that CNVs serve as distinct targets for breeding strategies across different geographical contexts. A similar assumption was made in a recent study on minipig populations targeting demographic signatures of selection [94].

For example, we identified CNVRs in Ossabaw and Yucatan minipigs associated with lipid metabolism. This was highlighted by an overlap of genes in these regions enriched for ascorbate and aldarate metabolism and QTL linked to intramuscular fat content, stearic acid content, and subcutaneous fat thickness. This finding might hint at the extremely increased ability of the Ossabaw minipig to convert food into body fat and its outstanding propensity to obesity and the development of metabolic syndrome [97, 98]. Similarly, the Yucatan minipig was studied as a model for obesity for the effects of a low-calorie/low-protein diet on weight and body composition [99]. This breed’s predisposition to obesity might be further supported by our findings of genes enriched in CNVRs, overlapping with the Hippo signalling pathway, which is known to control the balanced regulation of adipocyte proliferation and differentiation [100] and was previously tied to meat quality traits [101].

In contrast to the American breeds, negative regulation of Hippo signalling was enriched in common CNVRs shared by the breeds from Asia and Oceania (Kune Kune, Vietnamese Potbellied, and Wuzhishan). This pathway might play a role in the specific phenotypic expression of the different breed types, mainly as Hippo signalling is relevant for cell proliferation, as mentioned above, and is also highly important for organ size control [102]. Furthermore, the identified QTL “litter weight” and “teat number” for this group of pigs, as well as the enrichment of genes in exclusive CNVRs for the oxytocin signalling pathway, likely reflect the efforts to increase breeding efficiency for conservation purposes [103]. In addition, early sexual maturity and good adaptability to harsh rearing conditions or poor nutrition were highlighted as unique characteristics common in these breeds [103–107].

Similar considerations apply to European minipig breeds (Aachen Minipig, Göttingen Minipig, Mini-LEWE), where QTL for traits such as “teat number” and “sperm abnormality rate” were found to overlap with exclusive CNVRs, indicating selection to refine reproductive attributes. We propose that these similarities regarding reproduction might be due to the breeding history of these minipigs, which resulted from the crossbreeding of Vietnamese potbellied pigs with commercial farm pigs in Europe to develop miniature-sized pigs for biomedical research [106, 108]. Additional exclusive CNVRs coincided with QTL “body height” and “shoulder weight,” suggesting an association with breed-specific selection for reduced body size [108–110]. In addition, a large number of enriched genes were associated with the cardiovascular system, including “angiogenesis”, “cardiac muscle cell action potential”, and “abnormal myocardial fibre morphology”, potentially reflecting the strong emphasis on cardiac research in European minipigs [111, 112]. It was proposed that the minipig’s extensive similarities to the human heart in anatomy and physiology make it an attractive alternative to traditional non-rodent species for cardiac and respiratory safety studies [111, 113]. Studies for cardiovascular safety pharmacology have highlighted the significantly longer cardiac action potentials and QT intervals (time between the start of the Q wave and the end of the T wave) of the (Göttingen) minipig compared to dogs at comparable cycle lengths as well as a relative resistance to arrhythmias [114]. Research on xenografts and immunological safety frequently involved Göttingen minipigs [115], which aligns with our findings of QTL for “CD3-negative” and “CD8-positive/negative leukocyte percentage” in CNVRs, presumably reflecting artificial selection for immunological traits [116, 117].

Overall, our CNVR findings in European minipigs and other geographically diverse breeds spotlight essential features tied to structural variation in desirable genes and genomic regions under selection in miniature pigs [93], including body size, conformation, meat quality, and disease susceptibility [68, 118]. Incorporating CNV data into breeding programmes can help maintain genetic diversity while improving desirable traits, as demonstrated in breeds such as the Aachen Minipig and Göttingen minipig [93, 108].

Nevertheless, gene set enrichment studies require cautious interpretation since they focus on coding regions and rely on existing gene annotations. In recent years, it has been highlighted that structural variants in non-coding DNA have modifying or pathogenic potential, affecting the spatial 3D organisation of the genome and leading to gene dosage effects [119, 120]. Future investigations should include CNVs in non-coding regions to provide a broader perspective on phenotypic impacts, particularly in growth control or disease susceptibility traits.

## Conclusions

This study provides a comprehensive view of CNVs in miniature pig breeds and advances our understanding of how geographic distribution impacts genetic diversity. The results reveal distinctive patterns shaped by targeted breeding strategies in pigs used for biomedical research purposes. This study sheds light on the complex CNV landscape as a valuable resource for future studies in pigs and improves the genetic understanding of breeding strategies and their impact on structural variation as footprints of artificial selection in livestock populations.

## Methods

### Sampling and whole genome sequencing

This study utilised WGS data from 36 miniature pig samples representing nine different miniature pig breeds from various locations worldwide, as detailed in Additional File 1: Table S1. In total, 20 samples were obtained from a previous study [121] (project identifier PRJNA795885) or downloaded directly from the National Center for Biotechnology Information (NCBI)-Sequence Read Archive (SRA). Ethical approval for these samples can be found in the respective publications.

In addition, further hair roots, ear cartilage (from ear tagging) or skin samples (from two piglets that died due to perinatal mortality) were collected from 16 minipigs, including three Kune Kune, four Aachen Minipig, three Mini-Lewe, three Potbellied, and three other miniature pig breeds, and underwent WGS. Subsequently, DNA was extracted from non-degraded tissues to minimise any confounding impact on genomic integrity.

Sample preparation followed the protocol described in our previous study [121], adhering to all national and international guidelines. All samples were sequenced on an Illumina NovaSeq 6000 platform, generating 150 bp paired-end reads to ensure high accuracy and resolution. The animal experiments were approved by the animal ethics committee of the Lower Saxony State Veterinary Office (registered at 33.9-42502-05-17A217). All sampled pigs were owned by the Institute of Animal Genomics. Informed consent for using the samples was obtained from the pig owner. Due to the low burden of sampling, no anaesthesia was applied. This global data collection ensured the broad applicability of our findings to diverse genomic research and animal breeding contexts.

### WGS analysis

Raw sequencing reads were filtered using the fastp v. 0.23.4 with specific parameters to improve data integrity. These parameters included adapter trimming to remove sequencing adaptors, low complexity filtering to reduce artefacts *(--low_complexity_filter --complexity_threshold 1*), and quality trimming to ensure high-quality reads *(--cut_front --cut_front_window_size 1 --cut_front_mean_quality 20 and --cut_tail --cut_tail_window_size 1 --cut_tail_mean_quality 20*). Furthermore, bases were filtered based on a Phred score threshold *(--qualified_quality_phred 15*), with reads discarded if more than 70% of the bases were of low quality (*--unqualified_percent_limit 70*) or if the number of N bases exceeded 50 *(--n_base_limit 50*).

Filtered reads were aligned to the autosomal reference genome *Sus scrofa 11.1* [36] accessed from Ensembl, using the Burrows-Wheeler alignment (BWA) tool v. 0.7.17-r1188 [122]. The parameters -t 8 (for multithreading) and -M (to mark shorter split hits as secondary) were used to ensure proper handling of split alignments. Coverage and depth estimates were calculated using samtools v. 1.19.2 (command samtools depth) [123], averaging the read depth across all autosomal bases with mapped reads. Multiple CNV detection tools were employed to cross-validate CNVs, thereby reducing false positives in lower-depth regions and minimising coverage-related biases.

Duplicates were marked, and the processed data were indexed using Picard tools v. 3.1.0 [124]. Each sample’s data quality was re-evaluated to ensure that low-depth or high-coverage regions would not disproportionately impact CNV detection.

### CNV detection and analysis

Following prior studies’ recommendations, four tools were used to detect CNVs, providing a multi-tool approach to optimise CNV recall and precision [5, 59]. All tools were applied exclusively to autosomes to eliminate sex-related biases in CNV analysis.

Lumpy [125], integrated via Smoove v. 0.2.8, leveraged split-read signals [126]. Default parameters were applied, specifying a minimum variant size of 50 bp (*--min-event-size 50*) and genotypes were jointly called to obtain a population-wide CNV dataset. Furthermore, Delly v. 1.1.8 [127] was applied for a comprehensive analysis combining paired-end and split-read data with default settings, specifying -z 50 as the minimum CNV size. Genome Analysis Toolkit (GATK) gCNV pipeline v. 4.5 relied on read-depth signals to detect CNVs, using a bin size of 1000 as recommended for 30x data to balance detection rates and false positive control [128]. Finally, CNVpytor v. 1.3.1 analysed CNVs based on read depth and allele imbalance [129], using a bin size of 500 bp following the recommendations for approximately 37x coverage and 150 bp reads [129]. All tools employed normalisation procedures (for GC content and local coverage variation) to reduce systematic biases. CNVs larger than 5 Mb were excluded to limit the number of false-positive rates due to repetitive regions or assembly uncertainties, as previously suggested [130].

Nine pig breeds were examined for differences in population structure using the CNVs pooled from the four CNV datasets. All CNVs were combined into a union dataset; duplicates were removed by merging CNVs of the same type using a minimum 50% reciprocal overlap criterion with GenomicRanges v. 1.56.1 [131] in R 4.3.3 [132]. A binary matrix was then constructed indicating the presence or absence of CNVs per sample. Principal component analysis for population structure analysis was performed using multidimensional scaling with the cmdscale function in R 4.3.3 [132] and visualised as a scatterplot using ggplot2 [133]. A neighbour-joining tree was constructed using a genetic distance matrix to assess phylogenetic relationships between the studied populations, which were visualised using MEGA v. 11 [134].

To further investigate variants with a higher likelihood of a functional impact, we extracted homozygous deletions (copy number state 0, potential for gene disruption) and homozygous duplications (copy number state ≥ 4, prone to gene dosage imbalances) present in all 36 minipig samples from all CNV calling datasets for further analysis. These CNVs were merged using a minimum 50% reciprocal overlap criterion with GenomicRanges v. 1.56.1 [131] in R 4.3.3 [132]. Subsequently, the merged CNV dataset intersected with all exonic regions extracted from the Ensembl *Sus scrofa 11.1* genome annotation (release 111). All CNVs with at least 50% overlap with one or more exons were retained. Corresponding genes were assigned to these exonic regions, and gene annotations were performed using Ensembl biomaRt v. 2.60.1 [135, 136].

### Real-time quantitative PCR

Seven CNVs within exonic regions (see analysis above) and a random selection of five CNVs across different chromosomes were validated using real-time quantitative PCR. In total, five gains (*EEA1*,* GLYAT*,* SUSD4*,* CYB5R1*, *TRMT6*) and seven losses (*CYP4A24*,* SCAND3*,* PLCD4*,* RICTOR*,* SPATC1*, *COL6A1– two regions*) were examined using isolated DNA from seven miniature pigs also used for sequencing and further two additional minipigs (Additional file 6: Table S4). All qPCR assays were designed following the TaqMan copy number assay guidelines (Thermo Fisher Scientific, Waltham, MA, USA). As a reference, the genomic region covering *CSK (C-terminal src kinase)* was used, which has not been reported to exhibit any CNVs in porcine studies so far but was found to be copy-number stable in the human genome [137, 138]. Reactions were prepared in triplicates, with each 20 μl multiplex reaction containing 20 ng DNA, 1X TaqPath ProAmp MasterMix (Thermo Fisher Scientific, Waltham, MA, USA), 1X assay (FAM dye), and 1X reference assay (VIC dye). qPCR was run on a QuantStudio 3 system (Applied Biosystems, Foster City, CA, USA) using a standard TaqMan copy number assay protocol: 95 °C for 10 min, followed by 40 cycles of 95 °C for 15 s and 60 °C for 60 s. The data were analysed using CopyCaller software v2.1 (Thermo Fisher Scientific, Waltham, MA, USA), and relative copy numbers were estimated based on the assumption of deltaCt = 0 representing the diploid state.

### CNV region detection and analysis

For each of the four CNV datasets, homozygous and heterozygous CNVs, including deletions (loss), duplications (gain), and any CNVs with higher copy numbers, were assembled into CNVRs. CNVs overlapping by at least one base pair were merged into CNVRs using the populationRanges function in CNVRanger v. 1.20.0 [139] in R 4.3.3 [132]. Genomic regions of low density (less than 10% of the total contributing individual calls within a combined region) were removed from the CNVRs to avoid false positives. The CNVRs were divided into deleted ‘loss’ and duplicated ‘gain’ regions, while overlapping ‘gain’ and ‘loss’ regions were combined into a single region categorised as ‘both’.

Next, overlapping CNVRs of the same type across the four tool-specific datasets were combined if they met the mutual one bp overlap criterion and appeared in at least two methods. CNVRs in all nine miniature pig breeds were identified by retaining those found in at least two individuals per breed. Regional CNVRs were defined by grouping the breeds (America, Asia & Oceania, and Europe) and retaining CNVRs present in every animal from that region. Singletons (CNVs present in only one individual) and doubletons (present in two individuals) were recorded but not included in the consensus CNVRs to ensure robustness. The distribution of CNVs across individuals and breeds is summarised in Additional File 2: Table S2.

The distribution of CNVRs was visualised as a bar plot using ggplot2 [133] and, additionally, with gene density, as an ideogram using RIdeogram v. 0.2.2 [140] in R 4.3.3 [132]. Gene density was calculated in 1-Mb windows based on the *Sus scrofa 11.1* genome annotation. Chromosomal coordinates were retrieved from assembly metadata. CNVRs were plotted as overlays on gene density data to highlight regions of interest.

### Enrichment of genes and quantitative trait loci overlapping CNVRs

Genome annotation was obtained from Ensembl (release 111), and pig QTL was retrieved from the animal QTL database [141] for the *Sus scrofa 11.1* reference genome. An overlap of at least one bp between CNVRs and genomic features (genes or QTL) was used to identify potentially affected regions. A Fisher’s exact test [142] was performed on all QTL overlapping with CNVRs to determine whether specific QTL were overrepresented compared to their overall genome distribution in R 4.3.3 [132]. The complete pig QTL dataset in the animal QTL database served as background references.

Functional enrichment analysis was performed by mapping pig genes to human orthologues using gprofiler2 v. 0.2.3 [143], then conducting enrichment analyses with enrichR v. 3.2 [144, 145] in R 4.3.3 [132], drawing upon “GO_Biological_Process_2023” [146–148], “KEGG_2021_Human” [149–151], “MGI_Mammalian_Phenotype_Level_4_2021” [152–154] and “PhenGenI_Association_2021” [155]. All terms with a *p*-value < 0.05 were considered significant. All Ensembl IDs were reviewed to remove duplicates from overlapping annotations in multi-copy CNVRs and avoid overrepresentation.

### Analysis of exclusive CNVRs between different geographic regions

Non-overlapping, exclusive CNVRs specific to America, Asia & Oceania, or Europe were identified by comparing CNVRs from one region with those in other regions using GenomicRanges v. 1.56.1 [131] in R 4.3.3 [132]. Exclusive CNVRs did not overlap with CNVRs in other regions; for these region-specific CNVRs, associated genes and QTL were extracted, converting Ensembl to Entrez IDs with Ensembl biomaRt v. 2.60.1 [135, 136]. Subsequent functional annotation and biological theme comparisons were performed using ClusterProfiler v. 4.12.3 [156, 157] with the *Sus scrofa 11.1* reference genome in R 4.3.3 [132]. This analysis included GO biological processes and KEGG pathway enrichment to determine the biological themes represented by the genes overlapping with the exclusive CNVRs of each region. Finally, QTL linked to these exclusive CNVRs were summarised according to functional categories such as “growth and development”, “reproduction”, “meat and carcass quality”, and “health and immunity”, offering insights into region-specific trait–association.

## Electronic supplementary material

Below is the link to the electronic supplementary material.

Supplementary Material 1

Supplementary Material 2

Supplementary Material 3

Supplementary Material 4

Supplementary Material 5

Supplementary Material 6

Supplementary Material 7

Supplementary Material 8

Supplementary Material 9

Supplementary Material 10

Supplementary Material 11

Supplementary Material 12

Supplementary Material 13

Supplementary Material 14

## Data Availability

All WGS data are available on the NCBI SRA (PRJNA635602, https://www.ncbi.nlm.nih.gov/Traces/study/?acc=PRJNA635602).

## Abbreviations

aCGH: Array-based Comparative Genomic Hybridisation
BWA: Burrows-Wheeler Alignment
CD36: CD36 molecule, thrombospondin receptor
CELSR1: Cadherin EGF LAG seven-pass G-type receptor 1
CHD1L: Chromodomain helicase DNA binding protein 1
CHD4: Chromodomain helicase DNA binding protein 4
CIT: Citron Rho-interacting serine/threonine kinase
CNOT9: CCR4-NOT transcription complex subunit 9
CNVRs: Copy number variant regions
CNVs: Copy number variants
COL6A1: Collagen type VI alpha 1 chain
CSK: C-terminal Src Kinase
CYP3A29: Cytochrome P450 family 3 subfamily A member 29
CYP4A24: Cytochrome P450 family 4 subfamily A member 24
EEA1: Early endosome antigen 1
EIF4A2: Eukaryotic translation initiation factor 4A2
ELOVL6: ELOVL fatty acid elongase 6
FISH: Fluorescence in situ hybridisation
GATK: Genome Analysis Toolkit
GO: Gene Ontology
GWAS: Genome-wide association study
INDEL: Insertion or deletion
KEGG: Kyoto Encyclopaedia of Genes and Genomes
MDS: Multidimensional scaling
MP: Mammalian Phenotype
NCBI: National Center for Biotechnology Information
NJ: Neighbour-joining
PAG6: Pregnancy-associated glycoprotein 6
PCA: Principal component analysis
PCR: Polymerase chain reaction
PLCD4: Phospholipase C delta 4
qPCR: Real-time quantitative PCR
QTL: Quantitative trait loci
RICTOR: RPTOR independent companion of MTOR complex 2
RLN: Relaxin family peptide hormone
SCAND3: SCAN domain containing 3
SNP: Single nucleotide polymorphism
SRA: Sequence Read Archive
SUSD4: Sushi domain containing 4
U6: U6 spliceosomal RNA
WGS: Whole genome sequencing
