# Supplementary material for: Multi-tool copy number detection highlights common body size-associated variants in miniature pig breeds from different geographical regions
Source: BMC Genomics. 2025 Mar 22;26:285. doi: 10.1186/s12864-025-11446-8 (PMC11929999; doi:10.1186/s12864-025-11446-8)

**Additional file 3: Figure S1.**

Format: tif

Title: **CNV length distribution by detection tool for small (< 10 kb) and large (≥ 10 kb) CNVs.**

Description: Histograms illustrating the distribution of CNV lengths identified by each tool (CNVPytor, Delly, GATK gCNV, Smoove) are shown separately for small CNVs (< 10 kb) (a) and large CNVs (≥ 10 kb) (b). The bin size for panel (a) is 500 bp, while for panel (b), it is 5,000 bp.


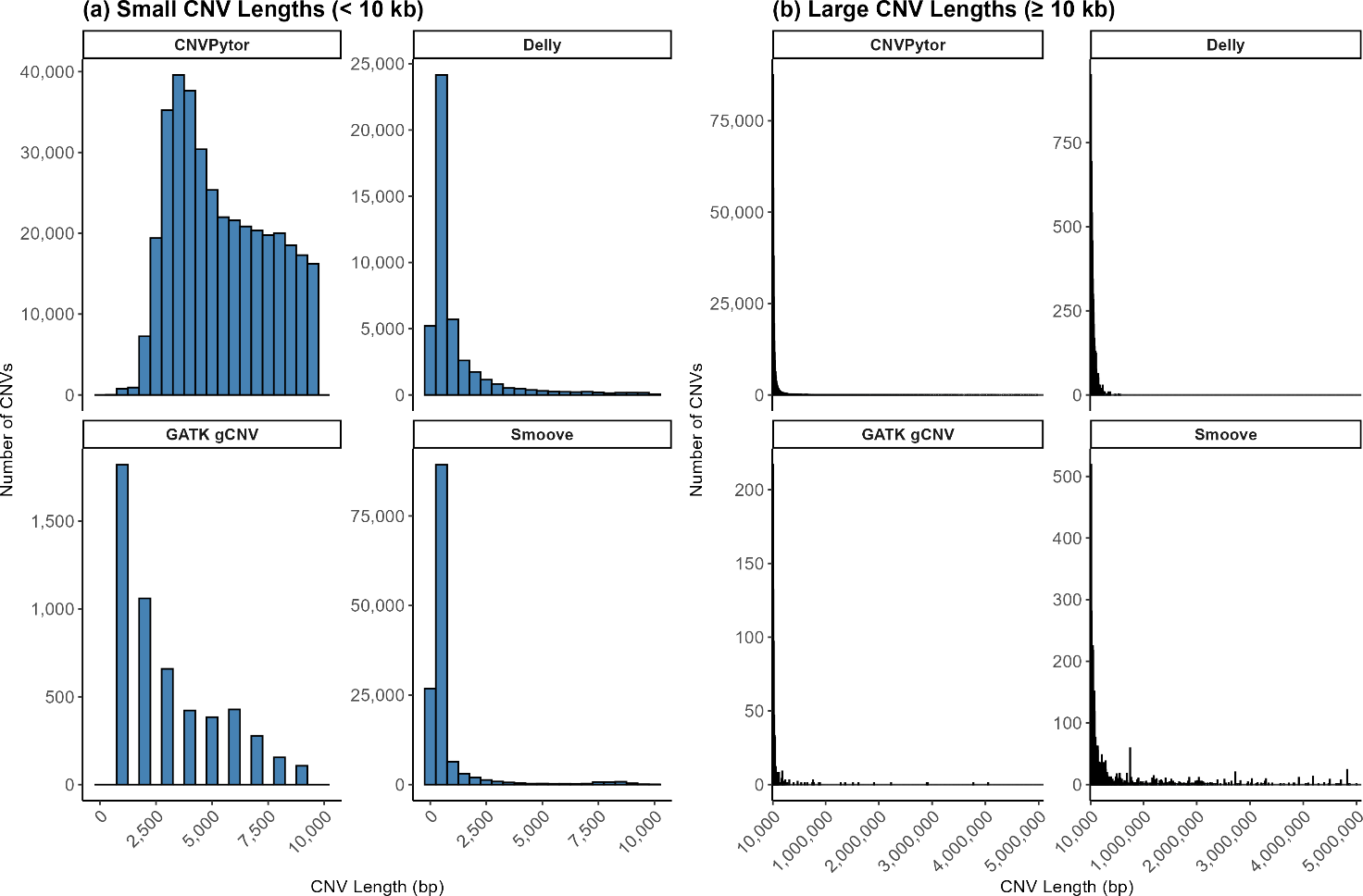

Supplement: Supplementary file 3 — Supplementary Material 3 [file 12864_2025_11446_MOESM3_ESM.docx]
