# Supplementary material for: Multi-tool copy number detection highlights common body size-associated variants in miniature pig breeds from different geographical regions
Source: BMC Genomics. 2025 Mar 22;26:285. doi: 10.1186/s12864-025-11446-8 (PMC11929999; doi:10.1186/s12864-025-11446-8)

**Additional file 4: Figure S2.**

Format: tif

Title: **Population structure of miniature pig populations based on CNV data.**

Description: (a) Multidimensional scaling (MDS) plot visualising the population structure of nine miniature pig populations based on CNV data. Each point represents an individual, with colours and symbols corresponding to specific breeds. (b) Neighbour-joining (NJ) tree constructed based on CNV data illustrating the phylogenetic relationships among the nine populations.


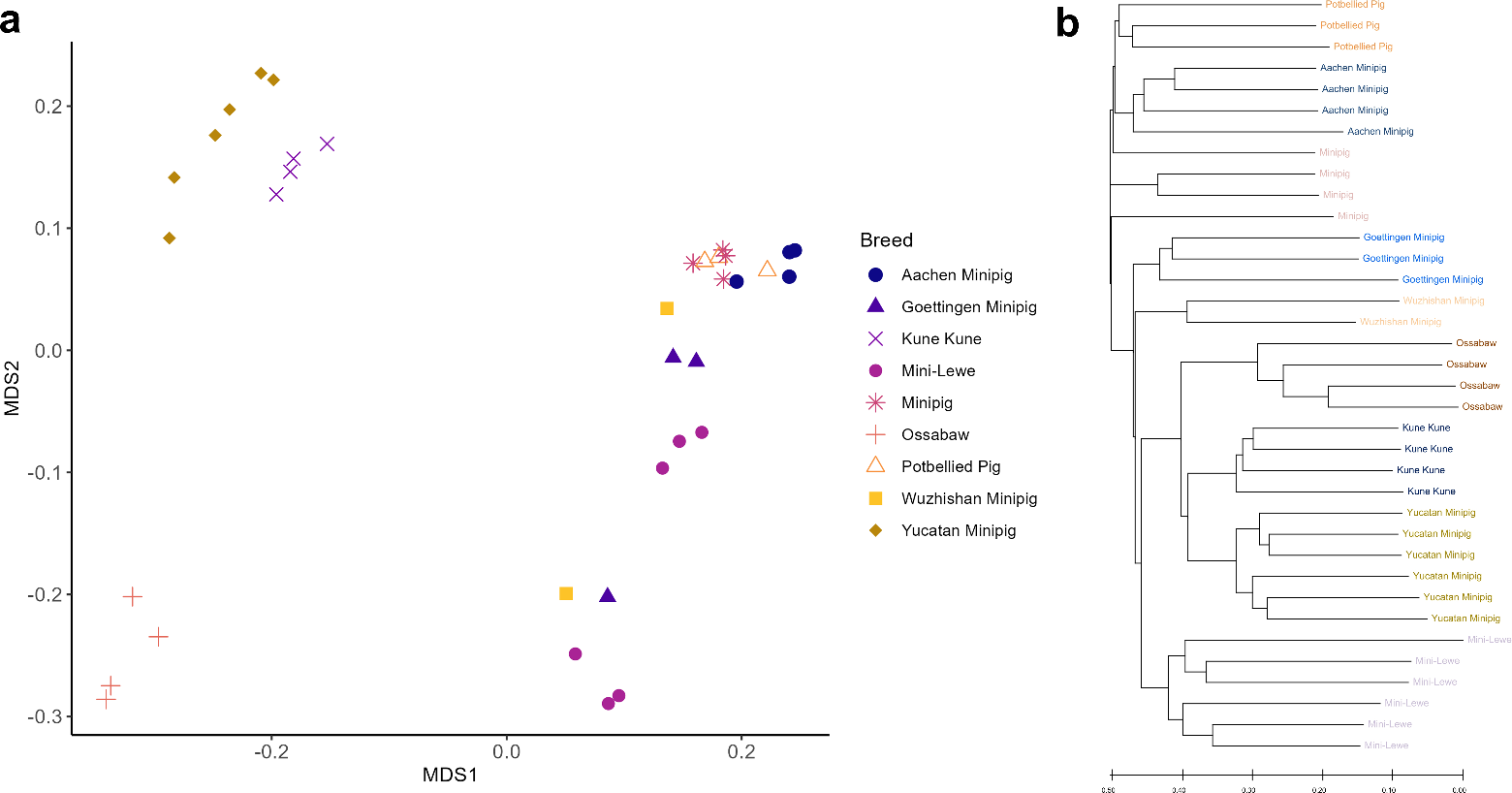

Supplement: Supplementary file 4 — Supplementary Material 4 [file 12864_2025_11446_MOESM4_ESM.docx]
