# Supplementary material for: Multi-tool copy number detection highlights common body size-associated variants in miniature pig breeds from different geographical regions
Source: BMC Genomics. 2025 Mar 22;26:285. doi: 10.1186/s12864-025-11446-8 (PMC11929999; doi:10.1186/s12864-025-11446-8)

**Additional file 8: Figure S3.**

Format: tif

Title: **Ideograms depicting the distribution of shared CNVRs across all nine global and regional minipig-breed groups.**

Description: Background bars represent the 18 pig autosomes, with gene density visualized as a heat map (red shading indicates higher density). Green triangles indicate deleted (‘loss’) CNVRs, blue boxes highlight duplicated (‘gain’) CNVRs, and orange circles represent regions with ‘loss’ and ‘gain’ (‘both’) CNVRs. The ideograms are organized into four panels: one for all minipig breeds combined (All Minipigs) and three for regional breed groups (America, Asia & Oceania, Europe).


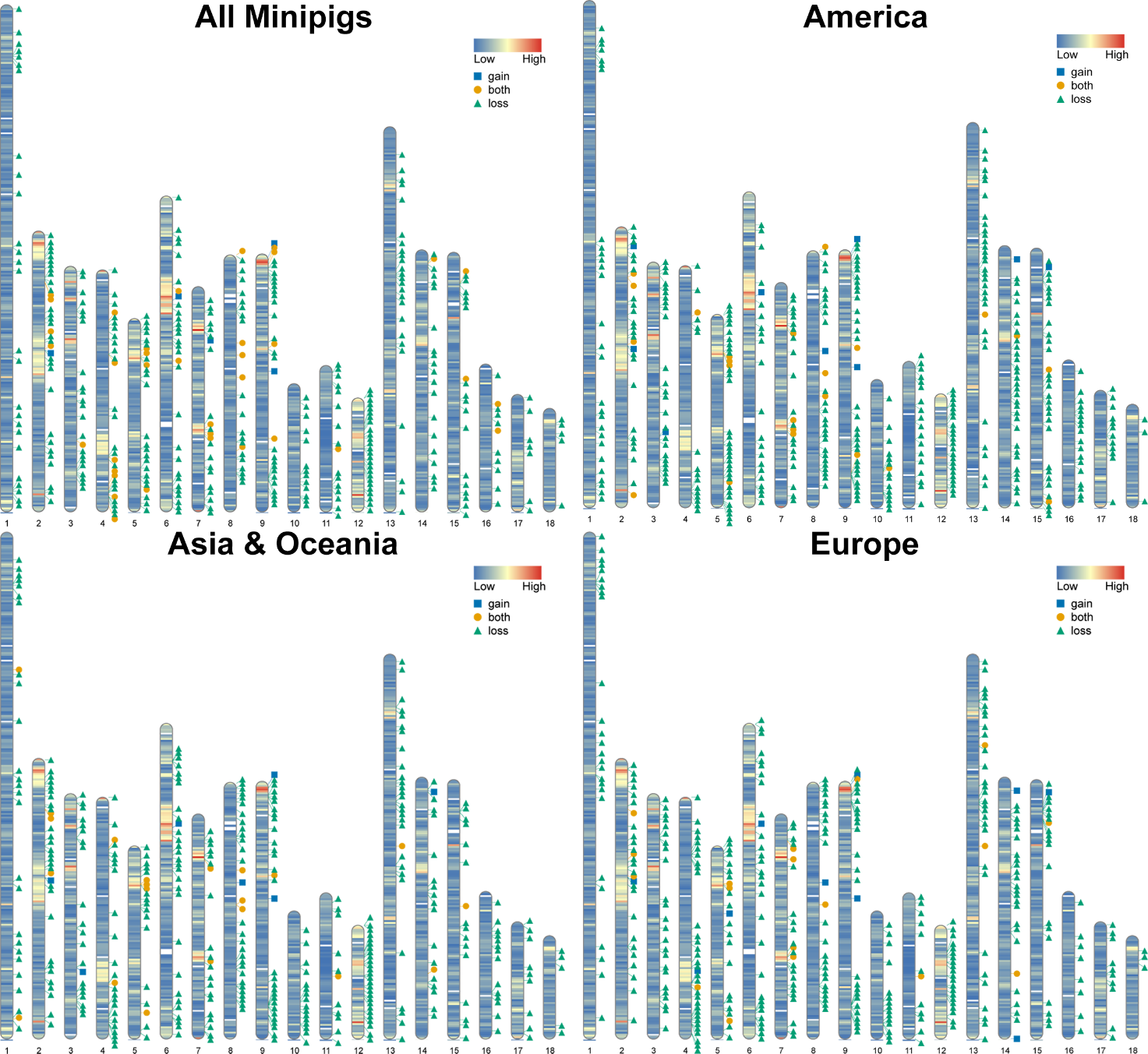

Supplement: Supplementary file 8 — Supplementary Material 8 [file 12864_2025_11446_MOESM8_ESM.docx]
