# Supplementary material for: Multi-tool copy number detection highlights common body size-associated variants in miniature pig breeds from different geographical regions
Source: BMC Genomics. 2025 Mar 22;26:285. doi: 10.1186/s12864-025-11446-8 (PMC11929999; doi:10.1186/s12864-025-11446-8)

**Additional file 9: Figure S4.**

Format: tif

Title: **Overview of CNVR length distribution according to CNVR type, geographic region (a), and CNVR distribution relative to chromosomal length (b).**

Description: The length distribution of CNVRs (in kilobase pairs, kb) for different CNVR types (all, loss, gain, both) and region-based groups are shown (a). The correlation factor between chromosomal length and CNVR number is at the top of the figure (b).


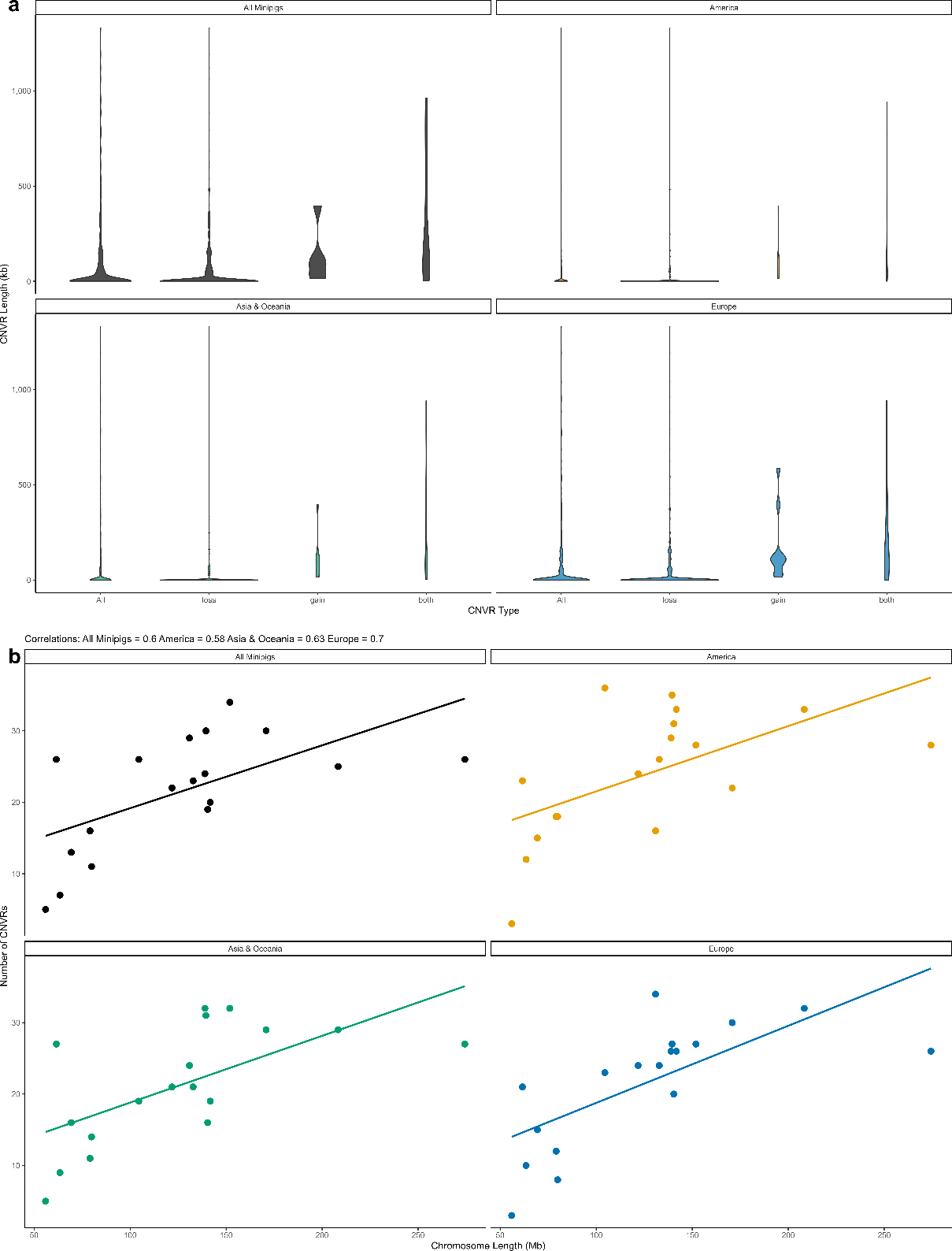

Supplement: Supplementary file 9 — Supplementary Material 9 [file 12864_2025_11446_MOESM9_ESM.docx]
